# Two risk factors for hypozincemia in diabetic β-thalassemia patients: Hepatitis C and deferasirox

**DOI:** 10.1371/journal.pone.0284267

**Published:** 2024-01-12

**Authors:** Hadi Darvishi-Khezri, Hossein Karami, Mohammad Naderisorki, Mobin Ghazaiean, Mehrnoush Kosaryan, Amir Mosanejad-Galchali, Aily Aliasgharian, Hasan Karami

**Affiliations:** 1 Thalassemia Research Center (TRC), Hemoglobinopathy Institute, Mazandaran University of Medical Sciences, Sari, Iran; 2 Gut and Liver Research Center, Non-communicable Disease Institute, Mazandaran University of Medical Sciences, Sari, Iran; 3 MD. Islamic Azad University, Sari Branch, Mazandaran, Iran; 4 Department of Pediatrics, Mazandaran University of Medical Sciences, Sari, Iran; Gachon University Gil Medical Center, DEMOCRATIC PEOPLE’S REPUBLIC OF KOREA

## Abstract

**Background and aim:**

Hypozincemia is a prevalent adverse consequence in diabetes mellitus (DM) and β-Thalassemia patients. We aimed to evaluate the level of serum zinc in β-thalassemia patients with DM and a risk assessment for hypozincemia.

**Methods:**

The study population included transfusion-dependent thalassemia (TDT) and non-transfusion-dependent thalassemia (NTDT) with overt DM (fasting plasma glucose (FPG) ≥126 mg/dL, and/or 2-h plasma glucose≥200 mg/dL). Serum zinc concentration was measured by the colorimetric method, and the values below 70 μg/dL were defined as hypozincemia. Myocardial and liver T2*-weighted magnetic resonance imaging (MRI T2*, millisecond [ms]) were valued by a free contrast MRI. The demographic, clinical, paraclinical, and laboratory data were also recorded. The data belonged to the period from December 2018 until December 2020.

**Results:**

Of 64 diabetic β-thalassemia patients, 41 cases had zinc data in their medical files (aged 38 ± 9 years, 48.8% female). 78.05% of patients (n = 32) were TDT, and 21.95% were NTDT (n = 9). The mean ± standard deviation of zinc level was 110.2 ± 127.6 μg/dL. The prevalence of hypozincemia was 9.76%, 95% confidence interval [CI] 0.27 to 19.24 (four cases). After controlling age, the odds of hypozincemia for using deferasirox (DFX) was 8.77, 95% CI 0.60 to 127.1. In β-thalassemia patients, the age-adjusted risk of hypozincemia was calculated at 15.85, 95% CI 0.47 to 529.3 for hepatitis C. The adjusted risk of hypozincemia based on age for antacid use was 6.34, 95% CI 0.39 to 102.7.

**Conclusion:**

In light of this study, as well as hepatitis C, using DFX and antacids is associated with a high risk of hypozincemia amid diabetic β-thalassemia cases. However, upward bias should be taken into consideration.

## Introduction

β-Thalassemia is a group of heterogeneous disorders inherited in the form of autosomal recessive. The disease is classified according to the type of defect in the β-globin chain, characterizing insufficient erythropoiesis and iron overload [1]. Some factors, including the age of receiving a red blood cell (RBC) transfusion, the age of beginning iron chelation therapy, iron siderosis in vital organs (e.g., liver, cardiac, and endocrine glands), tolerance to chelation therapies, and red-cell transfusion requirements have been considered as the disease intensity in these cases [2,3]. According to the continuous glucose monitoring system (CGMS), the highest incidence of diabetes mellitus (DM) has been reported in patients with a β-thalassemia major in their second and third decades [4].

Zinc, as a vital element, in addition to its role in pancreatic cells, also plays a pivotal role in improving the control of blood glucose in peripheral tissues [5]. Serum zinc concentration regulates blood sugar, insulin storage and secretion, and RBC survival [6]. Zinc deficiency (hypozincemia) is found in three to 30% of β-thalassemia patients. An increased zinc requirement [7] and hyperzincuria ensuing from iron chelation therapy, hyperferritinemia, and liver dysfunction can be enumerated as the underlying causes of hypozincemia in thalassemic cases.

Insulin resistance and damaging beta cells caused by an iron overload may play a significant role in emerging impaired glucose homeostasis (IGH) in patients with β-thalassemia [8,9]. Moreover, such determinations as the frequency of red-cell transfusion, and treatment with iron chelators, for instance, deferoxamine, can impact the blood glucose homeostasis in these patients [10]. Therefore, this study planned to evaluate serum zinc levels in β-thalassemia patients with DM and perform a risk assessment for hypozincemia.

## Method

### Ethics statement

The study proposal and protocol were verified by the Vice Chancellor of Research and Technology, the Ethics Committee, and the Institutional Review Board of Islamic Azad University of Sari (no IR.IAU.SARI.REC.1399.024, date 2020-05-02). All patients got informed and became aware by all physicians and nurses working with these patients before data registration. In the current study, informed written consent was taken from all the participants. All cases were assured that their information would be confidentially kept.

### Study design and population

The study protocol was established at Thalassemia Research Center (TRC), Bu Ali Sina Hospital, Sari. The current research was a cross-sectional study in which diabetic β-thalassemia patients entered. According to the results of the first pretransfusion complete blood count (CBC), hemoglobin electrophoresis, and genetic test, β-thalassemia cases were identified by their accountable physicians. The study population included transfusion-dependent thalassemia (TDT) and non-transfusion-dependent thalassemia (NTDT), referred to the thalassemia ward at Bu Ali Sina Hospital. The diabetic β-thalassemia cases with serum zinc data were recruited in the study. Based on another inclusion criterion, the cases with overt DM entered the study. The diagnosis of DM was based on American Diabetes Association (ADA) criteria: FPG ≥ 126 mg/dL (7.0 mmol/L) and/or 2-h plasma glucose (2hPG) ≥ 200 mg/dL (11.1 mmol/L) [11]. Defecting the existing patient’s information was an exclusion criterion.

### Data collection

At the thalassemia ward, an experienced staff recorded the cases’ information, including demographic, clinical, paraclinical, and laboratory findings. In the current study, data belonged to the period from December 2018 to December 2020. The data encompassed age, sex, body mass index (BMI), dependency on red-cell transfusion, the length of blood transfusions, iron chelation therapies, splenectomy, time since splenectomy, and co-morbidities such as hepatitis C, chronic kidney disease (CKD), chronic liver disease (CLD) and peptic ulcer disease (PUD). Biological tests findings comprised the mean of serum ferritin levels in the study period, bilirubin profile (direct, indirect, and total), the average of the three recent fasting blood sugars (FBS) during the study timeframe, alanine aminotransferase (ALT), aspartate aminotransferase (AST) and alkaline phosphatase (ALP). Some therapeutic data, such as using zinc supplementation, the dose of zinc supplementation (mg/day), agents suppressing gastric acid secretion—antacids, histamine type 2 blockers (H2 blockers), and proton pump inhibitors (PPIs)—metformin and insulin were also extracted from patients’ medical files.

### Serum zinc measurement

Serum zinc concentration was measured by the colorimetric method, and 5-Br-PAPS [2-(5-Brom-2-pyridyl azo)-5-[N-propyl-N-(3-sulfopropyl)-amino]-phenol Dinatriumsalz Dihydrat] as a reagent (Biorexfars ZINC kit, Iran; limit of detection [LOD], five μg/dl). The normal range of serum zinc level was 120 to 170 μg/dL, and values below 70 μg/dL were itemized as hypozincemia [12].

### Magnetic resonance imaging (MRI)

T2*-weighted magnetic resonance imaging (MRI T2*, millisecond [ms]) was valued by a free contrast MRI performed on a 1.5 T MR scanner (Symphony; Siemens, Germany). The Brompton protocol was applied for performing cardiac MRI [13]. The midpoint of the liver was scanned at 12 various echo times—1.29 to 23 ms, increasing in 2.2 ms increments—employing a single trans-axial slice. The myocardial and liver MRI T2* results were considered a reduced value in the case of myocardial MRI T2* <20 ms and liver MRI T2* <6.3 ms, signifying a cardiac and liver hemosiderosis [14]. Liver iron concentration (LIC, mg Fe/g dry liver tissue) was also obtained, thereby the liver MRI.

### Statistical analysis

All statistical procedures were performed by the SPSS software package (version 20.0, SPSS Inc., Chicago, IL, USA) and STATA version 13 (StataCorp, College Station, TX, USA). Data have been reported as mean ± standard deviation (M±SD) or number (percent). We applied the Mann-Whitney U and Fisher’s exact tests to compare the variables between two groups, hypozincemia versus non-hypozincemia. The correlation between serum zinc level and age was estimated using the Spearman correlation coefficient. The determining factors were evaluated by estimating the odds ratio for hypozincemia. The effects of multiple potential confounders, including age and duration of using zinc supplementation, were controlled to calculate the adjusted odds ratio. A logistic regression model was eventually utilized to sort the risk factors based on their strength in this study. The goodness-of-fit of the model was assessed using the Hosmer and Lemeshow test. For the final model, multicollinearity between independent variables was also tested by the LMCOL command, based on the VIF index (variance inflation factor). The estimated odds ratios (ORs) were converted to risk ratios (RRs) to reduce the overestimation bias (https://clincalc.com/Stats/ConvertOR.aspx). With a 95% confidence interval, the prevalence of hypozincemia was also approximated using the exact binomial method by STATA 13 software. We ultimately used a resampling approach by running the Jackknife method to unveil the effects of small sample size on our results. A P-value lower than 0.05 was chosen as the threshold of statistical significance.

## Results

Of 64 diabetic β-thalassemia patients, 41 cases had zinc data in their medical file (aged 38 ± 9 years, 48.8% female). 78.05% of patients (n = 32) were TDT, and 21.95% were NTDT (n = 9). The mean zinc level was 110.2 ± 127.6 μg/dL [median 89.0, first quartile 78, third quartile 105 μg/dL]. The prevalence of hypozincemia and hyperzincemia was 9.76%, 95% CI 0.27 to 19.24 (four cases) and 2.44%, 95% CI -2.49 to 7.39 (one case), respectively. The basic and clinical characteristics of diabetic β-thalassemia patients, alongside their laboratory and paraclinical information, have been shown in Tables 1 and 2.

**Table 1:** Basic and clinical characteristics of diabetic β-thalassemia patients.

Variable		Hypozincemia, zinc level<70 μg/dL		P-value
		yes (n = 4)	no (n = 37)	
Age, year		32.7 ± 8.25	33.1 ± 9.16	0.13 ^ƒ^
Gender	Male	2 (50)	19 (51.35)	0.95 ^ǃ^
	Female	2 (50)	18 (48.65)	
BMI, kg/m^2^		20 ± 4	22 ± 3	0.40 ^ƒ^
Red-cell transfusion dependency		3 (75)	29 (78.38)	0.88 ^ǃ^
Length of red-cell transfusion, year		32.6 ± 8.52	30.62 ± 9.01	0.42 ^ƒ^
Duration of DM, year		13.6 ± 6.4	11.2 ± 6.6	0.39 ^ƒ^
Splenectomy		4 (100)	27 (72.97)	0.23 ^ǃ^
Duration past after splenectomy, year		33.9 ± 4.9	25.7 ± 8.4	0.16 ^ƒ^
PUD		-	3 (13.64)	0.57 ^ǃ^
Chronic liver disease		1 (25)	-	**0.004** ^ǃ^
CKD		-	2 (6.25)	0.66 ^ǃ^
Hepatitis C		1 (25)	3 (8.82)	0.32 ^ǃ^
DFO		3 (75.00)	31 (83.78)	0.66 ^ǃ^
DFX		2 (50.00)	6 (16.22)	0.10 ^ǃ^
DFP		-	2 (5.41)	0.63 ^ǃ^
Combination therapy with iron chelators		3 (75.00)	26 (70.27)	0.84 ^ǃ^
Zinc supplementation		3 (75.00)	27 (81.82)	0.74 ^ǃ^
Dose of zinc supplementation, mg/day		16.7 ± 2.9	17.3 ± 8.5	0.60 ^ƒ^
Duration of using zinc supplementation, year		14 ± 6.93	9.54 ± 6.33	0.30 ^ƒ^
Insulin		4 (100)	27 (72.97)	0.23 ^ǃ^
Duration of using insulin, year		18.2 ± 5.4	12.7 ± 6.1	0.21 ^ǃ^
Metformin		1 (25.00)	5 (13.51)	0.54 ^ǃ^
Antacids ^a^		2 (50.00)	11 (29.73)	0.41 ^ǃ^

BMI: Body mass index, DM: Diabetics multiuse, PUD: Peptic ulcer disease, CKD: Chronic kidney disease, DFO: Deferoxamine, DFX: Deferasirox, DFP: Deferiprone.

Data are presented as mean ± standard deviation or number (%).

^a^ Agents suppressing gastric acid secretion, including antacids, histamine type 2 blockers, and proton pump inhibitors.

ǃ P-value was obtained by Fisher’s exact test.

ƒ P-value was calculated by the Mann-Whitney U test.

**Table 2:** Laboratory and paraclinical information of diabetic β-thalassemia patients.

Variable	Hypozincemia, zinc level<70 μg/dL		P-value
	yes (n = 4)	no (n = 37)	
Ferritin, ng/mL	1878 ±1071	2579 ± 1874	0.60
ALT, IU/L	24.25 ± 12.17	36.21 ± 25.15	0.63
AST, IU/L	31.5 ±17.37	31.88 ± 16.26	0.49
ALP, IU/L	315.33 ± 114.03	258.6 ± 117.6	0.34
Bilirubin direct, mg/dL	0.38 ±0.17	0.37 ± 0.22	0.46
Bilirubin indirect, mg/dL	0.83 ± 0.62	1.13 ± 1.01	0.59
Bilirubin total, mg/dL	1.48 ±1.24	1.51 ± 1.10	0.52
FBS, mg/dl	192 ±74	189 ± 76	0.46
LIC, mg Fe/g dry liver tissue	4.73 ±2.37	7.11 ± 6.34	0.59
Liver MRI T2*, ms	6.39 ± 4.70	7.23 ± 6.48	0.49
Myocardial MRI T2*, ms	26.31 ± 11.34	19.27 ± 8.22	0.24

ALT: Aspartate aminotransferase, AST: Alanine aminotransferase, ALP: Alkaline phosphatase, FBS: Fasting blood sugar, LIC: Liver iron concentration, ms: Millisecond

MRI T2*: T2-weighted magnetic resonance imaging.

Data are presented as mean ± standard deviation or number (%).

^a^ Agents suppressing gastric acid secretion, including antacids, histamine type 2 blockers, and proton pump inhibitors.

P-value was calculated by the Mann-Whitney U test.

The correlation between serum zinc level and age was approximated at -0.39; P = 0.01. An increase in the length of DM and using insulin were associated with the risk of hypozincemia (OR 1.94, 95% CI 0.29 to 12.6 and OR 5.18, 95% CI 0.76 to 34.53, correspondingly). A risk for hypozincemia was also found with an increase of serum ALP, OR 2.40, 95% CI 0.36 to 15.68.

A crude odds of 5.17, 95% CI 0.60 to 44.18, was estimated for hypozincemia in the cases under deferasirox (DFX) therapy. After controlling age, the OR of hypozincemia for using DFX was 8.77, 95% CI 0.60 to 127.1. Following converting ORs to RRs index, the modified risk of hypozincemia for patients under treatment with DFX changed to 21% (RR 1.21 95% CI 0.88 to 1.24).

The crude odds of hypozincemia in the cases affected by hepatitis C were obtained at 3.44, 95% CI 0.27 to 44.32. In β-thalassemia patients, the age-adjusted risk of hypozincemia was calculated at 15.85, 95% CI 0.47 to 529.3 for hepatitis C. The adjusted risk of 11% was also found for hypozincemia in cases with hepatitis C, RR 1.11, 95% CI 0.89 to 1.12. A crude odds of 2.36, 95% CI 0.29 to 18.97, was computed for hypozincemia in those who received antacids treatment. The age-adjusted risk of hypozincemia for antacid use based on age was 6.34, 95% CI 0.39 to 102.7. The risk of hypozincemia, 36%, rose in those who took antacids after a bias correction (RR 1.36, 95% CI 0.67 to 1.46).

The crude odds of hypozincemia in the cases with liver MRI T2* lower than 6.3 ms was calculated at 1.47, 95% CI 0.12 to 17.91. The adjusted risk of hypozincemia in patients with reduced liver MRI T2* (<6.3 ms) (< 10 ng/mL) has been valued at 3.07, 95% CI 0.09 to 109.1, after fixing the effects of age and length of using zinc supplementation. A 65% boost in the risk of hypozincemia was computed for patients with reduced liver MRI T2* (<6.3 ms) (RR 1.65, 95% CI 0.14 to 2.37). The potential determinant factors for hypozincemia in β-thalassemia patients have been displayed in Table 3. After resampling by the Jackknife method, all the estimated ORs did not change. The results of resampling (Jackknife method) have been displayed in Table 4.

**Table 3:** Potential determinant factors for hypozincemia (< 70 μg/dL) in diabetic β-thalassemia patients (n = 41).

Variable	OR with 95% CI			
	Model ^1^	Model ^2^	Model ^3^	Model ^4^
DFX	5.17, 0.60 to 44.18	8.77, 0.60 to 127.1	6.77, 0.44 to 104.5	7.48, 0.40 to 139.5
Hepatitis C	3.44, 0.27 to 44.32	15.85, 0.47 to 529.3	2.89, 0.15 to 56.65	8.38, 0.17 to 408.9
Antacids ^a^	2.36, 0.29 to 18.97	6.34, 0.39 to 102.7	2.31, 0.14 to 36.59	2.69, 0.12 to 59.41
Reduced liver MRI T2*, <6.3 ms	1.47, 0.12 to 17.91	1.57, 0.09 to 26.93	1.81, 0.07 to 47.81	3.07, 0.09 to 109.1
Reduced myocardial MRI T2*, <20 ms	0.5, .041 to 6.05	0.64, 0.03 to 12.81	-	-

OR: Odds ratio, CI: Confidence interval, DFX: Deferasirox

MRI T2*: T2-weighted magnetic resonance imaging, ms: Millisecond.

^a^ Agents suppressing gastric acid secretion, including antacids, histamine type 2 blockers, and proton pump inhibitors.

**Model** ^**1**^: Unadjusted odds ratios.

**Model** ^**2**^: ORs were adjusted according to age.

**Model** ^**3**^: ORs were adjusted according to the duration of using zinc supplementation.

**Model** ^**4**^: ORs were adjusted according to age and duration of using zinc supplementation.

**Table 4:** The results of resampling (Jackknife method).

Variable	OR with 95% CI			
	Model ^1^	Model ^2^	Model ^3^	Model ^4^
DFX	5.17, 0.27 to 96.77	8.77, 0.06 to 1269.4	6.77, 0.50 to 91.94	7.48, 0.06 to 858.1
Hepatitis C	3.44, 0.48 to 24.59	15.85, 0.24 to 1036.9	2.89, 0.20 to 41.25	8.38, 0.42 to 167.3
Antacids ^a^	2.36, 0.13 to 41.53	6.34, 0.02 to 2317.3	2.31, 0.14 to 38.32	2.69, 0.02 to 383.4
Reduced liver MRI T2*, <6.3 ms	1.47, 0.19 to 11.34	1.57, 0.09 to 26.64	1.81, 0.11 to 29.24	3.07, 0.07 to 142.2
Reduced myocardial MRI T2*, <20 ms	0.5, 0.06 to 3.82	0.64, 0.04 to 10.34	-	-

OR: Odds ratio, CI: Confidence interval, DFX: Deferasirox

MRI T2*: T2-weighted magnetic resonance imaging, ms: Millisecond.

^a^ Agents suppressing gastric acid secretion, including antacids, histamine type 2 blockers, and proton pump inhibitors.

**Model** ^**1**^: Unadjusted odds ratios.

**Model** ^**2**^: ORs were adjusted according to age.

**Model** ^**3**^: ORs were adjusted according to the duration of using zinc supplementation.

**Model** ^**4**^: ORs were adjusted according to age and duration of using zinc supplementation.

The multivariate logistic regression analysis revealed that the risk of hypozincemia for using DFX surged to 15.81, 95% CI 0.56 to 446.8. An increase in the risk of hypozincemia for hepatitis C was also obtained, OR 7.11, 95% CI 0.18 to 275.1. Moreover, the risk of hypozincemia for using antacids and reduced liver MRI T2* (<6.3 ms) changed to 4.35, 95% CI 0.17 to 108.9 and 2.67, 95% CI 0.06 to 108.9, correspondingly. The multivariate logistic regression model results have been presented in Table 5.

**Table 5:** The results of multivariate logistic regression analysis for hypozincemia (< 70 μg/dL) in diabetic β-thalassemia patients (n = 41).

Variable	SE	Wald	Adjusted OR, 95% CI	P-value
DFX	1.86	2.62	15.81, 0.56 to 446.8	0.10
Hepatitis C	1.64	1.10	7.11, 0.18 to 275.1	0.29
Antacids ^a^	1.89	0.80	4.35, 0.17 to 108.9	0.37
Reduced myocardial MRI T2*, <20 ms	1.70	0.28	2.72, 0.06 to 115.2	0.60
Reduced liver MRI T2*, <6.3 ms	1.91	0.27	2.67, 0.06 to 108.9	0.60
Constant	3.13	0.001	0.003, 0.0001 to 0.90	**0.04**

OR: Odds ratio, CI: Confidence interval, SE: Standard error, DFX: Deferasirox

MRI T2*: T2-weighted magnetic resonance imaging, ms: Millisecond.

The logistic regression model was not statistically significant, *X* ^2^ (5, n = 41) = 6.056, P = 0.30. Log-likelihood was 14.05, and the model explained between 16.8% (Cox & Snell *R*^*2*^) and 36.7% (Nagelkerke *R*^*2*^) of the variance in hypozincemia (< 70 μg/dL) and also correctly classified 90.9% of cases. The goodness of fit for the model was appropriate (Hosmer and Lemeshow χ2 = 5.34, P = 0.62).

## Discussion

In this study, around 10 percent of diabetic β-thalassemia suffered from zinc deficiency. In our center, administering zinc supplementation led to conserving zinc levels in the optimum ranges. Therefore, prescribing zinc supplementation at the right dose and duration can significantly help correct serum zinc levels, particularly in at-risk cases. Furthermore, the evaluation of the results indicated that using DFX was associated with hypozincemia risk, nearly 21%. In these cases, the risk of hypozincemia in diabetic patients with hepatitis C was 11%, compared to non-hepatitis C cases. The use of antacids was linked to a 36% risk of hypozincemia. Any grade of liver hemosiderosis was accompanied by a 65% risk of zinc deficiency. Although the small sample of our study resulted in non-significant associations between hypozincemia and the potential risk factors in these cases, the resampling approach revealed that the calculated ORs were not affected by the low sample size.

Previously, zincuria was reported as a frequent adverse consequence in patients with DM [15]. Increased urinary excretion of zinc can be the key driver for zincuria in thalassemia patients with DM [16]. A cross-sectional study [17] demonstrated that the rate of hypozincemia was equal to 100% in patients with β-thalassemia cases, which prevailed more in males. In a study [18], the prevalence of hypozincemia was reported to be 33% higher in men. Another study [19] also observed hypozincemia in 24% of 75 cases of β-thalassemia, which was 2-fold in men. The prevalence of hypozincemia in our study was 9.76% and was not predominant in either sex, contrary to the above studies.

Zinc deficiency may occur in β-thalassemia cases for various reasons, including constant red-cell transfusion, hyperferritinemia, liver hemosiderosis and dysfunction, urinary overexcretion, and higher demand for zinc—as cofactors of antioxidant enzymes—in response to raised oxidative stress attributable to hemolysis and iron overload [18,20]. In addition, hypozincemia tends to be presented as a repercussion of excessive zinc losses that stem from chronic hemolysis in this population [20]. The rigorous iron burden may damage hepatocytes under the surged oxidative stress levels by overproducing free radicals, which may more widely release zinc into the plasma [21]. As such, iron chelators can cause excessive zinc excretion following an increase in the extracellular zinc pool [22]. In β-thalassemia patients, the heme production process is where zinc element partakes to compensate for ineffective hematopoiesis, taking place based on anemia severity [23,24]. Furthermore, to regulate iron overload, some nutritional considerations restricting dietary iron-enriched sources, such as meats and cereals, are most often recommended for β-thalassemia cases, putting them at risk of trace elements deficiency, e.g., hypozincemia [24,25]. Moreover, to adhere to the transferrin binding sites competitively, the iron surplus cuts zinc absorption down in the gastrointestinal tract [26].

Thalassemia patients treated with iron chelators suffer from the chelation of zinc minerals, which is the most plausible mechanism of hypozincemia in these patients [26]. Other roles of iron chelators in the development of hypozincemia are related to the interaction of these medications with double bonds of zinc ions, decreasing serum zinc levels [27]. Moreover, iron chelators have a high affinity to zinc elements [28].

Deferasirox (Exjade, ICL670) is a potential factor in developing hypozincemia, increasing the chance of hypozincemia. Several studies have also pointed out that serum zinc levels in thalassemic cases under DFX therapy were lower than those treated with deferiprone (DFP) [22,27,29]. Based on the results of an investigation on 59 β-thalassemia major patients, although urinary zinc excretion after DFX therapy was significantly low compared to deferoxamine) DFO (plus DFP, the level of serum zinc was slightly less amid cases treated with DFX (64.8±14.8 μg/dL in DFX group versus 66.5±15.1 μg/dL in DFO plus DFP group). The urine levels of zinc in DFX group were 662.2±428.2 μg/day in comparison with 1182.3±980.3μg/day in DFO plus DFP group [26]. Consequently, zinc is lost in non-urinary ways, such as the gastrointestinal system, resulting in hypozincemia in this population.

The most excretion of zinc elements following DFX treatment occurs through biliary elimination via the gastrointestinal tract and feces [30]. A pharmacokinetics study [30] exhibited that the excretion of DFX in urine is minor. Only 6 to 11% of the administrated dose of DFX was excreted by urine in rats (mean: 101% of the dose, within seven days post-dose). 6.3, 69.3, and 21.5% of the administrated dose of DFX were recovered in urine, bile, and feces, respectively [30]. Besides, a disturbance in the zinc metabolism and homeostasis might tend to exist after DFX therapy, causing a lifted risk of hypozincemia in β-thalassemia patients, especially if the serum level of DFX peaks at the higher level of the therapeutic level [31,32].

Hepatitis C is another prominent factor in the development of hypozincemia. Nosheen Aslam et al. [33] conducted a case-control study involving patients with hepatitis C (HCV) (N = 25) and healthy control (N = 25), who examined the levels of heavy metals in the two groups. Their evaluation showed that the heavy metal levels were higher in hepatitis C cases. Serum zinc level in the HCV group was lower than healthy controls, indicating hypozincemia (42.9 ± 17.09 μg/dL versus 81.8 ± 13.52 μg/dL; P< 0.001) [33]. Chih-Hung Guo et al. [34] designed a study made up of three groups of patients, HCV (n = 30), HCV plus non-alcoholic fatty liver disease (NAFLD) (n = 32), and healthy controls (n = 30). They reported that serum zinc levels in the HCV group were lower compared to the healthy group (median 0.68 mg/L first quartile 0.65, third quartile 0.77 versus median 0.90 mg/L first quartile 0.77, third quartile 1.03), while the values were greater than HCV+NAFLD (median 0.58 mg/L first quartile 0.52, third quartile 0.63 in HCV+NAFLD group). The correlation estimated for serum ferritin levels and zinc concentrations was -0.60, presenting a moderate strength of association between them [34].

In HCV, due to liver damage caused by chronic inflammation and oxidative stress, heavy metal homeostasis is out of regulation, associated with increased accumulation [35]. The accumulation of metals such as copper (Cu) and lead (Pb) in these patients will be alongside the disruption of zinc homeostasis and hypozincemia [36]. Iron overload is of paramount importance to liver injury following HCV, which is mediated by cytotoxicity, immune-related processes, inflammation, and chronic oxidative stress [37]. Hepatotoxicity induced by iron overload alters zinc metabolism and causes zinc deficiency [38]. Another mechanism for the progression of hypozincemia in HCV patients would be the poor absorption of zinc, despite the zinc deficiency [39].

Prescription of gastric acid secretion inhibitors increases the risk of zinc deficiency. Serum zinc levels were assessed in a study (n = 46), including patients with gastroesophageal reflux disease who had a history of more than six months of use of PPIs [40]. The results demonstrated that the patients treated with PPIs had significant hypozincemia compared to non-users. In conformity with our study, their results indicated an almost 4-fold ramp up in serum zinc levels in non-users than the case group [40].

Using PPI might reduce the reabsorption of divalent ions, such as calcium, zinc, and magnesium by ascending the potential of hydrogen (PH) levels and descending the chloride content of the duodenum [41]. PPIs irreversibly inhibit the K-H pump of parietal cells, which deters the production of hydrochloric acid (HCL). The absence of gastric acid can cause significant problems in the absorption of nutrients and pathogens’ elimination [42,43].

Physiologically, zinc trace element plays a crucial role in multiple functions concerning glucose hemostasis, not only in the pancreas but also in insulin performance. Some evidence supports the connection between hypozincemia and the presence of insulin resistance in β-thalassemia patients [18,20]. To be precise, in β-thalassemia cases, the plasma zinc concentrations seem moderately correlated with β-cell function (*r* = 0.51) [44]. A pilot study on diabetic β-thalassemia patients (n = 9) unveiled that using zinc supplements for three months can bring an improvement in FPG to these cases. Accurately, a 3.7% fall in FPG was aligned with a 17% rise in plasma zinc concentration [44], representing the desired role of zinc element in improving glycemic metabolism. Intriguingly, the relationship between zinc intake and FPG was also shown across a meta-analysis carried out on non-thalassemic patients with type 2 DM (weighted mean difference [WMD] −20.34 mg/dL, CI −29.04 to −11.64), albeit the evidence level was not high [45]. Additionally, several studies have discovered the deleterious impact of inflammation and oxidative stress in emerging hyperinsulinemia, insulin resistance, and overt diabetes in β-thalassemia cases [46–48]. As well as anti-inflammatory properties [49] and stabilizing effects on RBCs [50], zinc can contribute to maintaining the structure of antioxidant enzymes, such as peroxidase, oxide dismutase, and catalase [49,51]. As such, the antioxidant effect of zinc would be positive in making IGH improve in β-thalassemia patients [6,52].

## Conclusion

As well as hepatitis C, using DFX and antacids is associated with a high risk of hypozincemia amid diabetic β-thalassemia cases, although overestimation bias should be viewed in this study. Meanwhile, a reduced myocardial and liver MRI T2* as cardiac and liver hemosiderosis can raise the risk of zinc deficiency in this population. The heterogenous β-thalassemia population in transfusion dependency was one of the limitations of the current study. Although the risk assessment revealed several risk factors according to the estimated effect sizes, the relationships were not significant due to the small sample size. Therefore, more studies should be carried out in the future to confirm the results. Ultimately, the results can have an overestimation bias, and readers should be cautiously aware once they interpret the results.

## Supporting information

S1 Data All data used in the analyses in this study are available as a supplementary file (STATA software). (DTA)

## Abbreviations

DM: diabetes mellitus
TDT: transfusion-dependent thalassemia
NTDT: non-transfusion-dependent thalassemia
FPG: fasting plasma glucose
MRI: magnetic resonance imaging
ms: millisecond
CI: confidence interval
DFX: deferasirox
RBC: red blood cell
CGMS: continuous glucose monitoring system
IGH: emerging impaired glucose homeostasis
IRB: Institutional Review Board
TRC: Thalassemia Research Center
CBC: complete blood count
ADA: American Diabetes Association
2hPG: 2-h plasma glucose
BMI: body mass index
CKD: chronic kidney disease
CLD: chronic liver disease
PUD: peptic ulcer disease
FBS: fasting blood sugars
ALT: alanine aminotransferase
AST: aspartate aminotransferase
ALP: alkaline phosphatase
H2 blockers: histamine type 2 blockers
PPIs: proton pump inhibitors
LIC: Liver iron concentration
VIF: variance inflation factor
ORs: odds ratio
SD: standard deviation
RR: risk ratio
DFP: Deferiprone
DFO: Deferoxamine
PH: potential of hydrogen
HCL: hydrochloric acid

## References

[1] Rubin R. New Gene Therapy for β-Thalassemia. Jama. 2022;328(11):1030–. 10.1001/jama.2022.14709 36125483

[2] Betts M, Flight PA, Paramore LC, Tian L, Milenković D, Sheth S. Systematic literature review of the burden of disease and treatment for transfusion-dependent β-thalassemia. Clinical Therapeutics. 2020;42(2):322–37. e2. 31882227 10.1016/j.clinthera.2019.12.003

[3] Yang G, Liu R, Peng P, Long L, Zhang X, Yang W, et al. How early can myocardial iron overload occur in beta thalassemia major? PloS one. 2014;9(1):e85379. doi: 10.1371/journal.pone.0085379 24465548 PMC3899006

[4] Soliman A, DeSanctis V, Yassin M, Elalaily R, Eldarsy NE. Continuous glucose monitoring system and new era of early diagnosis of diabetes in high risk groups. Indian journal of endocrinology and metabolism. 2014;18(3):274. doi: 10.4103/2230-8210.131130 24944918 PMC4056122

[5] Tang X-h Shay NF. Zinc has an insulin-like effect on glucose transport mediated by phosphoinositol-3-kinase and Akt in 3T3-L1 fibroblasts and adipocytes. The Journal of nutrition. 2001;131(5):1414–20. doi: 10.1093/jn/131.5.1414 11340092

[6] Matter RM, Elbarbary NS, Ismail EAR, Darwish YW, Nada AS, Banoub VP. Zinc supplementation improves glucose homeostasis in patients with β-thalassemia major complicated with diabetes mellitus: A randomized controlled trial. Nutrition. 2020;73:110702. 32007694 10.1016/j.nut.2019.110702

[7] Fung EB, Kwiatkowski JL, Huang JN, Gildengorin G, King JC, Vichinsky EP. Zinc supplementation improves bone density in patients with thalassemia: a double-blind, randomized, placebo-controlled trial. The American journal of clinical nutrition. 2013;98(4):960–71. doi: 10.3945/ajcn.112.049221 23945720 PMC3778866

[8] Dmochowski K, Finegood DT, Francombe W, Tyler B, Zinman B. Factors determining glucose tolerance in patients with thalassemia major. The Journal of Clinical Endocrinology & Metabolism. 1993;77(2):478–83. 10.1210/jcem.77.2.8345055 8345055

[9] Simcox JA, McClain DA. Iron and diabetes risk. Cell metabolism. 2013;17(3):329–41. doi: 10.1016/j.cmet.2013.02.007 23473030 PMC3648340

[10] Di Maggio R, Maggio A. The new era of chelation treatments: effectiveness and safety of 10 different regimens for controlling iron overloading in thalassaemia major. British journal of haematology. 2017;178(5):676–88. doi: 10.1111/bjh.14712 28439891

[11] Association AD. Diagnosis and classification of diabetes mellitus. Diabetes care. 2014;37(Supplement_1):S81–S90. doi: 10.2337/dc14-S081 24357215

[12] Smith J Jr, Butrimovitz G, Purdy W. Direct measurement of zinc in plasma by atomic absorption spectroscopy. Clinical Chemistry. 1979;25(8):1487–91. 455691

[13] Anderson L, Holden S, Davis B, Prescott E, Charrier C, Bunce N, et al. Cardiovascular T2-star (T2*) magnetic resonance for the early diagnosis of myocardial iron overload. European heart journal. 2001;22(23):2171–9. doi: 10.1053/euhj.2001.2822 11913479

[14] Darvishi-Khezri H, Karami H, Naderisorki M, Zahedi M, Razavi A, Kosaryan M, et al. Moderate to severe liver siderosis and raised AST are independent risk factors for vitamin D insufficiency in β-thalassemia patients. Scientific Reports. 2020;10(1):1–8. 33273639 10.1038/s41598-020-78230-4 PMC7712832

[15] Cunningham JJ, Fu A, Mearkle PL, Brown RG. Hyperzincuria in individuals with insulin-dependent diabetes mellitus: concurrent zinc status and the effect of high-dose zinc supplementation. Metabolism. 1994;43(12):1558–62. doi: 10.1016/0026-0495(94)90016-7 7990711

[16] Al-Refaie F, Wonke B, Wickens D, Aydinok Y, Fielding A, Hoffbrand A. Zinc concentration in patients with iron overload receiving oral iron chelator 1, 2-dimethyl-3-hydroxypyrid-4-one or desferrioxamine. Journal of clinical pathology. 1994;47(7):657–60. doi: 10.1136/jcp.47.7.657 8089225 PMC502110

[17] Mashhadi MA, Sepehri Z, Heidari Z, Shirzaee E, Kiani Z. The prevalence of zinc deficiency in patients with thalassemia in South East of iran, sistan and baluchistan province. Iranian Red Crescent Medical Journal. 2014;16(8). 10.5812/ircmj.6243 PMC4222021 25389495

[18] Fung EB, Gildengorin G, Talwar S, Hagar L, Lal A. Zinc status affects glucose homeostasis and insulin secretion in patients with thalassemia. Nutrients. 2015;7(6):4296–307. doi: 10.3390/nu7064296 26043030 PMC4488784

[19] Sadia S, Syed Mohammad I, Jamal Uddin K, Rozina Z, Asim K. Zinc status and its correlation with basic parameters in transfusion dependent thalassemic patients: a Pakistani perspective. Iranian Journal of Blood and Cancer. 2014;6(3):113–8.

[20] Mousa SO, Abd Alsamia EM, Moness HM, Mohamed OG. The effect of zinc deficiency and iron overload on endocrine and exocrine pancreatic function in children with transfusion-dependent thalassemia: a cross-sectional study. BMC pediatrics. 2021;21(1):1–9. 34686155 10.1186/s12887-021-02940-5 PMC8532363

[21] Yeni H, Yani FF, Izzah AZ, Lubis G. Relationship between serum ferritin and zinc levels in patients with major thalassemia. Paediatrica Indonesiana. 2019;59(3):144–9.

[22] Zekavat OR, Bahmanjahromi A, Haghpanah S, Ebrahimi S, Cohan N. The zinc and copper levels in thalassemia major patients, receiving iron chelation therapy. Journal of Pediatric Hematology/Oncology. 2018;40(3):178–81. doi: 10.1097/MPH.0000000000001102 29420373

[23] Ozturk Z, Genc GE, Gumuslu S. Minerals in thalassaemia major patients: An overview. Journal of Trace Elements in Medicine and Biology. 2017;41:1–9. doi: 10.1016/j.jtemb.2017.01.001 28347454

[24] Nimkarn N, Songdej D, Dumrongwongsiri O, Sirachainan N, Chuansumrit A, Group TS. Age as a major factor associated with zinc and copper deficiencies in pediatric thalassemia. Journal of Trace Elements in Medicine and Biology. 2021;68:126817. doi: 10.1016/j.jtemb.2021.126817 34298330

[25] Fung EB, Xu Y, Trachtenberg F, Odame I, Kwiatkowski JL, Neufeld EJ, et al. Inadequate dietary intake in patients with thalassemia. Journal of the Academy of Nutrition and Dietetics. 2012;112(7):980–90. doi: 10.1016/j.jand.2012.01.017 22551675 PMC3419338

[26] Erdoğan E, Canatan D, Örmeci AR, Vural H, Aylak F. The effects of chelators on zinc levels in patients with thalassemia major. Journal of Trace Elements in Medicine and Biology. 2013;27(2):109–11. doi: 10.1016/j.jtemb.2012.10.002 23164519

[27] Lachowicz JI, Nurchi VM, Crisponi G, de Guadalupe Jaraquemada-Pelaez M, Ostrowska M, Jezierska J, et al. Zinc (II) and copper (II) complexes with hydroxypyrone iron chelators. Journal of Inorganic Biochemistry. 2015;151:94–106. doi: 10.1016/j.jinorgbio.2015.08.011 26281974

[28] Genc GE, Ozturk Z, Gumuslu S, Kupesiz A. Mineral levels in thalassaemia major patients using different iron chelators. Biological trace element research. 2016;170:9–16. doi: 10.1007/s12011-015-0441-1 26179086

[29] Kusumawardhani W, Harsono Salimo MR. Plasma Zinc Difference in Children with Thalassemia β Major Received Deferiprone or Deferasirox Zinc. Indonesian Journal of Medicine. 2020;5(02):102–8.

[30] Bruin GJ, Faller T, Wiegand H, Schweitzer A, Nick H, Schneider J, et al. Pharmacokinetics, distribution, metabolism, and excretion of deferasirox and its iron complex in rats. Drug metabolism and disposition. 2008;36(12):2523–38. doi: 10.1124/dmd.108.022962 18775980

[31] Crisponi G, Nurchi VM, Crespo-Alonso M, Sanna G, Zoroddu MA, Alberti G, et al. A speciation study on the perturbing effects of iron chelators on the homeostasis of essential metal ions. PloS one. 2015;10(7):e0133050. doi: 10.1371/journal.pone.0133050 26192307 PMC4508027

[32] Karunaratna AMDS, Ranasingha JGS, Mudiyanse RM. Zinc status in beta thalassemia major patients. Biological trace element research. 2018;184:1–6. doi: 10.1007/s12011-017-1158-0 28940159

[33] Aslam N, Iqbal MS, Hussain SM, Rizwan M, Naseer Q, Afzal M, et al. Effects of chelating agents on heavy metals in Hepatitis C Virus (HCV) patients. Mathematical Biosciences and Engineering. 2019;16(3):1138–49. doi: 10.3934/mbe.2019054 30947412

[34] Guo C-H, Chen P-C, Ko W-S. Status of essential trace minerals and oxidative stress in viral hepatitis C patients with nonalcoholic fatty liver disease. International Journal of Medical Sciences. 2013;10(6):730. doi: 10.7150/ijms.6104 23630437 PMC3638296

[35] Iqbal MS, Ashfaq UA, Khaliq S, Masoud MS, Qasim M, Haque A, et al. Toll-like receptor 4 polymorphism as pretreatment predictor of response to HCV genotype 3a interferon-based treatment. Future Virology. 2017;12(12):739–46.

[36] Ozcelik D, Ozaras R, Gurel Z, Uzun H, Aydin S. Copper-mediated oxidative stress in rat liver. Biological trace element research. 2003;96:209–15. doi: 10.1385/BTER:96:1-3:209 14716100

[37] Simula MP, De Re V. Hepatitis C virus‐induced oxidative stress and mitochondrial dysfunction: A focus on recent advances in proteomics. Proteomics–Clinical Applications. 2010;4(10‐11):782–93. doi: 10.1002/prca.201000049 21137022

[38] Pramoolsinsap C, Promvanit N, Komindr S, Lerdverasirikul P, Srianujata S. Serum trace metals in chronic viral hepatitis and hepatocellular carcinoma in Thailand. Journal of Gastroenterology. 1994;29:610–5. doi: 10.1007/BF02365444 8000510

[39] Lin C-C, Huang J-F, Tsai L-Y, Huang Y-L. Selenium, iron, copper, and zinc levels and copper-to-zinc ratios in serum of patients at different stages of viral hepatic diseases. Biological trace element research. 2006;109:15–23. doi: 10.1385/BTER:109:1:015 16388099

[40] Farrell CP, Morgan M, Rudolph DS, Hwang A, Albert NE, Valenzano MC, et al. Proton pump inhibitors interfere with zinc absorption and zinc body stores. Gastroenterology Research. 2011;4(6):243. doi: 10.4021/gr379w 27957023 PMC5139861

[41] Sandström B, Abrahamsson H. Zinc absorption and achlorhydria. European Journal of Clinical Nutrition. 1989;43(12):877–9. 2697554

[42] Broeren MA, Geerdink EA, Vader HL, van den Wall Bake AWL. Hypomagnesemia induced by several proton-pump inhibitors. Annals of internal medicine. 2009;151(10):755–6. doi: 10.7326/0003-4819-151-10-200911170-00016 19920278

[43] Epstein M, McGrath S, Law F. Proton-pump inhibitors and hypomagnesemic hypoparathyroidism. New England Journal of Medicine. 2006;355(17):1834–6. doi: 10.1056/NEJMc066308 17065651

[44] Fung EB, Ahmad T, Killilea DW, Hussain R, Lal A. Zinc supplementation improves markers of glucose homeostasis in thalassaemia. British journal of haematology. 2020;190(3):e162–e6. doi: 10.1111/bjh.16771 32488893

[45] Wang X, Wu W, Zheng W, Fang X, Chen L, Rink L, et al. Zinc supplementation improves glycemic control for diabetes prevention and management: a systematic review and meta-analysis of randomized controlled trials. The American journal of clinical nutrition. 2019;110(1):76–90. doi: 10.1093/ajcn/nqz041 31161192

[46] Tangvarasittichai S, Pimanprom A, Choowet A, Tangvarasittichai O. Association of iron overload and oxidative stress with insulin resistance in transfusion-dependent beta-thalassemia major and beta-thalassemia/HbE patients. Clin Lab. 2013;59(7–8):861–8. doi: 10.7754/clin.lab.2012.120906 24133917

[47] Noetzli LJ, Mittelman SD, Watanabe RM, Coates TD, Wood JC. Pancreatic iron and glucose dysregulation in thalassemia major. American journal of hematology. 2012;87(2):155–60. doi: 10.1002/ajh.22223 22120775

[48] Muanprasat C, Wongborisuth C, Pathomthongtaweechai N, Satitsri S, Hongeng S. Protection against oxidative stress in beta thalassemia/hemoglobin E erythrocytes by inhibitors of glutathione efflux transporters. PloS one. 2013;8(1):e55685. doi: 10.1371/journal.pone.0055685 23383265 PMC3561311

[49] Fang Y-Z, Yang S, Wu G. Free radicals, antioxidants, and nutrition. Nutrition. 2002;18(10):872–9. doi: 10.1016/s0899-9007(02)00916-4 12361782

[50] Alaarg A, Schiffelers RM, van Solinge WW, Van Wijk R. Red blood cell vesiculation in hereditary hemolytic anemia. Frontiers in physiology. 2013;4:365. doi: 10.3389/fphys.2013.00365 24379786 PMC3862113

[51] Chasapis CT, Loutsidou AC, Spiliopoulou CA, Stefanidou ME. Zinc and human health: an update. Archives of toxicology. 2012;86:521–34. doi: 10.1007/s00204-011-0775-1 22071549

[52] Ferro FED, de Sousa Lima V, Soares NM, Cozzolino SF, do Nascimento Marreiro D. Biomarkers of metabolic syndrome and its relationship with the zinc nutritional status in obese women. Nutricion hospitalaria. 2011;26(3):650–4. doi: 10.1590/S0212-16112011000300032 21892588

